# Supplementary figures and images for: Prognostic significance of CD8+ T cell Spatial Biomarkers in ER+ and ER− breast cancer: A retrospective cohort study
Source: PLoS Med. 2025 Oct 15;22(10):e1004647. doi: 10.1371/journal.pmed.1004647 (PMC12539700; doi:10.1371/journal.pmed.1004647)

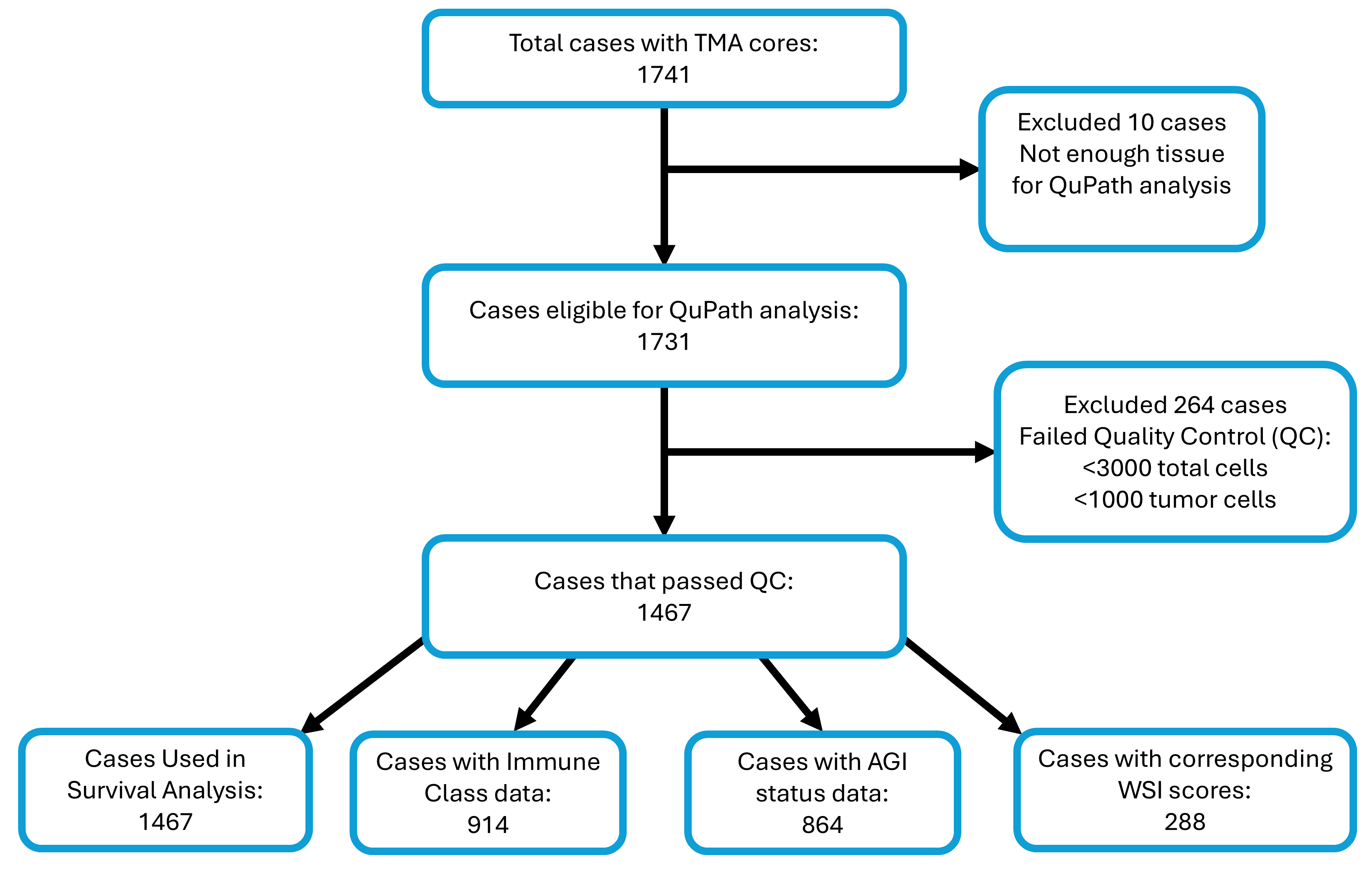

Supplement: S1 Fig — Diagram shows how many participants were excluded from the analysis due to quality control failures. Furthermore, the number of participants included in various analyses and with available WSI image and gene expression-derived classifications is presented. (PNG) [file pmed.1004647.s001.png]

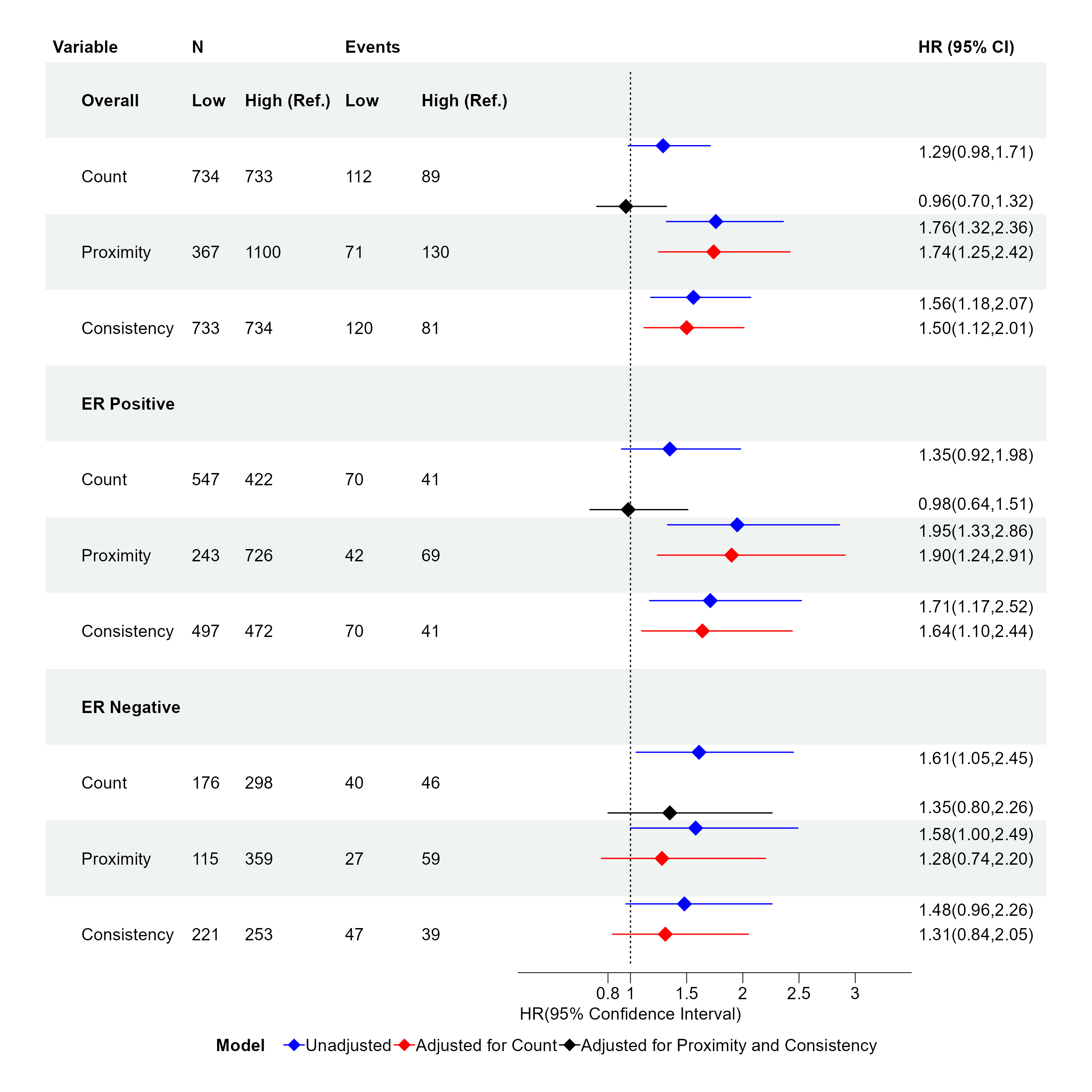

Supplement: S2 Fig — Hazard Ratios for Relapse Free Survival combining CD8+ /FoxP3− and CD8+/FoxP3+ lymphocytes to compute proximity and consistency. The hazard ratios (diamond) and 95% confidence intervals (lines) are shown for proximity, consistency, and lymphocyte count binarized at the median (high as referent), overall, and stratified by ER status. Estimates were unadjusted (blue), adjusted for lymphocyte count (red), and adjusted for both proximity and consistency (black) where relevant. The referent categories (Ref.) were defined as High Count, High Proximity, and High Consistency. Group size (N) and number of participants who experience recurrence (Event) are given for each group. HR: Hazard Ratio; 95% CI: 95% confidence interval. (PNG) [file pmed.1004647.s002.png]
