# Supplementary material for: Prognostic significance of CD8+ T cell Spatial Biomarkers in ER+ and ER− breast cancer: A retrospective cohort study
Source: PLoS Med. 2025 Oct 15;22(10):e1004647. doi: 10.1371/journal.pmed.1004647 (PMC12539700; doi:10.1371/journal.pmed.1004647)
Supplement: S1 Table — Table of tuning parameters for cell detection and thresholds for classification of cells used in QuPath functions. (DOCX) [file pmed.1004647.s003.docx]

| QuPath Cell detection Tuning Parameters | |
| --- | --- |
| Channel Used for Detection | Hoechst |
| Requested Pixel Size | 0.5 |
| Background Radius | 8 |
| Median Filter Radius | 0 |
| Sigma | 1.5 |
| Minimum Area | 10 |
| Maximum Area | 400 |
| Threshold | 11 |
| Cell Expansion | 3 |
| QuPath Classification Thresholds | |
| CD8 Positive Threshold | 25.12 |
| FoxP3 Positive Threshold | 80 |
| CK Positive Threshold | 31.62 |
