# Supplementary material for: Prognostic significance of CD8+ T cell Spatial Biomarkers in ER+ and ER− breast cancer: A retrospective cohort study
Source: PLoS Med. 2025 Oct 15;22(10):e1004647. doi: 10.1371/journal.pmed.1004647 (PMC12539700; doi:10.1371/journal.pmed.1004647)
Supplement: S2 Table — The results from optimizing the cut point for the high versus low count, proximity, and consistency. We split the data based on a 2/3 to 1/3 training and testing split. Furthermore, we used stratified sampling on the recurrence data to ensure there would be an adequate number of participants with recurrence in the training and testing sets. We find an optimal cut point based on the quantile of the data, which minimizes the p-value for the log-rank test. Though the cut points appear to perform well on the training set, in most cases, there appears to be overfitting based on very different hazard ratios in the testing data. Furthermore, many results which were statistically significant in the training data are no longer statistically significant in the testing data. (DOCX) [file pmed.1004647.s004.docx]

*^a^Training data consisted of a random sample of 2/3 of the data (n=978)*

*^b^Test data consisted of a random sample of 1/3 of the data (n=519)*

**Training ^a^**

**Testing**

**^b^**

**High (ref) vs. Low**

**HR**

**p**

**-**

**value**

**HR**

**p**

**-**

**value**

**overall**

Count

2.07

0.0001

1.65

0.0792

Proximity

1.9

0.0003

2.02

0.0044

Consistency

1.69

0.0101

1.06

0.8503

**ER Positive**

Count

3.19

3.35E

-

07

1.54

0.2393

Proximity

2.58

0.0001

1.87

0.0709

Consistency

2.3

0.0002

0.87

0.7256

**ER Negative**

Count

5.9

2.84E

-

06

1.46

0.307

Proximity

7.59

0.0009

2

0.1888

Consistency

5.63

0.001

2.8

0.0775
