# Supplementary material for: Prognostic significance of CD8+ T cell Spatial Biomarkers in ER+ and ER− breast cancer: A retrospective cohort study
Source: PLoS Med. 2025 Oct 15;22(10):e1004647. doi: 10.1371/journal.pmed.1004647 (PMC12539700; doi:10.1371/journal.pmed.1004647)
Supplement: S3 Table — (DOCX) [file pmed.1004647.s005.docx]

|  | Proximity | Consistency | Count |
| --- | --- | --- | --- |
| Proximity  Consistency | 1.0000  0.4717 | 0.4717  1.0000 | 0.5545  0.3261 |
|  |  |  |  |
| Count | 0.5545 | 0.3261 | 1.0000 |
