# Supplementary material for: Prognostic significance of CD8+ T cell Spatial Biomarkers in ER+ and ER− breast cancer: A retrospective cohort study
Source: PLoS Med. 2025 Oct 15;22(10):e1004647. doi: 10.1371/journal.pmed.1004647 (PMC12539700; doi:10.1371/journal.pmed.1004647)
Supplement: S4 Table — A summary of multivariate Cox proportional hazards models with proximity, consistency, and lymphocyte count binary covariates for all participants. Estimates for the covariate-adjusted hazard ratios are provided, as well as p-values for the significance of the hazard ratios. Models with nested covariates are compared using the likelihood ratio test, and p-values for the significance of the difference between compared nested models are provided. The reference groups for each of the covariates are high proximity, high consistency, and high lymphocyte count, respectively. (DOCX) [file pmed.1004647.s006.docx]

| Models | Covariates | Estimated  Hazard Ratio | Estimate p-value | p-value for Likelihood Ratio Test | | |
| --- | --- | --- | --- | --- | --- | --- |
|  |  |  |  | **Model 3** | **Model 4** | **Model 5** |
| 1 | Low proximity | 1.94 | 5.43e-06 | 0.016 | 0.88 |  |
| 2 | Low lymphocyte count | 1.29 | 0.071 |  | 6.33e-05 | 1.57e-05 |
| 3 | Low proximity  Low consistency | 1.77  1.43 | 0.00015  0.017 |  |  | 0.55 |
| 4 | Low lymphocyte count  Low proximity | 0.98  1.96 | 0.88  5.11e-05 |  |  | 0.013 |
| 5 | Low proximity  Low consistency  Low lymphocyte count | 1.85  1.45  0.91 | 0.00025  0.014  0.55 |  |  |  |
