# Supplementary material for: Prognostic significance of CD8+ T cell Spatial Biomarkers in ER+ and ER− breast cancer: A retrospective cohort study
Source: PLoS Med. 2025 Oct 15;22(10):e1004647. doi: 10.1371/journal.pmed.1004647 (PMC12539700; doi:10.1371/journal.pmed.1004647)
Supplement: S5 Table — (DOCX) [file pmed.1004647.s007.docx]

| **Covariates** | **Multivariate**  **HR (95% CI)** | **Multivariate p-value** | **Univariate**  **HR (95% CI)** | **Univariate**  **p-value** |
| --- | --- | --- | --- | --- |
| **Low Proximity** | 2.31 [1.25, 4,29] | 0.01 | 1.94 [1.46, 2.58] | <0.005 |
|  |  |  |  |  |
| **Low Consistency** | 1.57 [0.91, 2.71] | 0.1 | 1.62 [1.22, 2.15] | <0.005 |
|  |  |  |  |  |
| **Low Count** | 0.69 [0.36, 1.30] | 0.25 | 1.29 [0.98, 1.71] | 0.07 |
|  |  |  |  |  |
| **Intratumoral Strength** | 0.67 [0.36, 1.26] | 0.22 | 0.48 [0.30, 0.78] | <0.005 |
|  |  |  |  |  |
| **Intratumoral Forest** | 1.01 [0.47, 2.16] | 0.98 | 0.52 [0.29, 0.92] | 0.03 |
|  |  |  |  |  |
| **Intratumoral Desert** | 0.60 [0.31, 1.15] | 0.13 | 1.43 [0.84, 2.43] | 0.18 |
|  |  |  |  |  |
| **Peritumoral Strength** | 0.66 [0.43, 1.02] | 0.06 | 0.58 [0.42, 0.79] | <0.005 |
