# Supplementary material for: Prognostic significance of CD8+ T cell Spatial Biomarkers in ER+ and ER− breast cancer: A retrospective cohort study
Source: PLoS Med. 2025 Oct 15;22(10):e1004647. doi: 10.1371/journal.pmed.1004647 (PMC12539700; doi:10.1371/journal.pmed.1004647)
Supplement: S7 Table — (DOCX) [file pmed.1004647.s009.docx]

| **Covariates** | **Multivariate**  **HR (95% CI)** | **Multivariate p-value** | **Univariate**  **HR (95% CI)** | **Univariate**  **p-value** |
| --- | --- | --- | --- | --- |
| **Low Proximity** | 1.88 [1.25, 2.84] | <0.005 | 1.94 [1.46, 2.58] | <0.005 |
|  |  |  |  |  |
| **Low Consistency** | 1.42 [0.98, 2.06] | 0.06 | 1.62 [1.22, 2.15] | <0.005 |
|  |  |  |  |  |
| **Low Count** | 0.81 [0.53, 1.22] | 0.31 | 1.29 [0.98, 1.71] | 0.07 |
|  |  |  |  |  |
| **CD8 T-Cell Signature** | 0.92 [0.78, 1.07] | 0.28 | 0.84 [0.73,0.96] | 0.01 |
