# Supplementary material for: Prognostic significance of CD8+ T cell Spatial Biomarkers in ER+ and ER− breast cancer: A retrospective cohort study
Source: PLoS Med. 2025 Oct 15;22(10):e1004647. doi: 10.1371/journal.pmed.1004647 (PMC12539700; doi:10.1371/journal.pmed.1004647)
Supplement: Attachment — Submitted filename: Response_to_Reviewer_Comments_PMEDICINE-D-25-01829R1_AEW.docx [file pmed.1004647.s013.docx]

We would like to thank the reviewers and editor for their time, as well as the opportunity to revise our manuscript. Reviewer comments, as well as editorial comments, are listed in *italics* with our responses below each comment. Numbering and section names within each Reviewer section correspond to the point-by-point format of comments in that review. Responses and text excerpts are given in blue. Replies to specific and general editorial comments are given after responses to all reviewers.

Reviewer #1

1.) *[MINOR] Please double check that all abbreviations are defined the first time they are used e.g., "IF" in the Abstract, "ER" in the Introduction, "TMA" used in Study Population before being defined in TMA Staining and Processing.*

**Reply:** We have double checked that abbreviations are defined the first time they are used. ER is defined in the abstract, and definitions for IF in the abstract and TMA in the methods have been added.

2.)	*[MINOR] Consider incorporating very brief definitions of "proximity" and "consistency" in the abstract. I understand word count makes this tricky, but these are the main innovation being presented.*

**Reply:** Definitions for “proximity” and “consistency” have been added to the abstract as follows: “Proximity and consistency are functions of the mean and variance of nearest neighbor distance between Ck+ tumor cells and CD8+ T-cells.”

3.)	*The authors say "Very few studies have evaluated proximity or other spatial characteristics and few have used specific cell-type markers in analyses… such as those available via multiplex immunofluorescent." but don't yet convincingly demonstrate this is the case. A quick search indicates there are more recent publications on this topic than those cited by the authors. Suggest more clearly establishing the gap in the existing literature by citing and succinctly critiquing the few available study examples and, if possible, citing relevant reviews that support the need for their research*.

**Reply:** We added additional citations from more recent papers and established the gap between their work and ours. We revised the discussion to reflect these new manuscripts (page 19, line 431):

Two recent studies considered spatial parameters of CD8 T cells [14,15]. One study established that density of CD8 T cells within 20 μm of breast cancer tumors is predictive of pathologically complete response and disease free survival in neoadjuvant therapy[15]. However, their methods still emphasized cell density (albeit within a defined range) as opposed to using distance to measure spatial differences. Moreover, they did consider survival differences when stratifying by cancer subtypes. The other study considered proximity of CD8 T cells to pancreatic ductal adenocarcinoma cells (PDAC) and observed longer overall survival when CD8+ T cells were closer to focal adhesion kinase (FAK)^+^ tumor cells. Inconsistencies in associations with ER+ disease in the previous literature may reflect unmeasured parameters or measurement of a factor that is correlated with an unknown metric; our data suggests that proximity and consistency are two plausible underlying parameters.

4.) *[MINOR] In the Introduction the authors talk generally about "mean distance" between tumour and immune cells, however they clarify later it's specifically nearest neighbour. Suggest tweaking wording to make this clear from the start.*

**Reply:** We have clarified in the introduction that “proximity” and “consistency” are based on the nearest neighbor distance between Ck+ tumor cells and CD8+ T-cells (page 5, line 94):

We considered proximity (measure of mean nearest neighbor distance (NND) between CD8+ and Ck+ tumor cells) and consistency (variability in NND between CD8+ and Ck+ tumor cells) as two novel spatial metrics describing the distribution of CD8+ T-cells within the TIME.

5.) *Given the cascade of participant exclusions presented under "Study Population" and the varying samples for various analyses, consider including a CONSORT-style figure that represents this visually.*

Reply: We have included a CONSORT-style diagram as Supplemental Figure 2.

7.) *[MINOR] The authors chose to consider a cell that exceeds the thresholds for both CK and CD8 or FoxP3 as a lymphocyte because "A cell cannot be both a tumor cell and a lymphocyte…". What was the reason for this decision? Note I am not suggesting this is incorrect - this is well outside my expertise - just that I am interested in the reasoning. The authors could also consider including a reference to support their decision.*

**Reply:** We wanted to ensure that we capture all relevant lymphocytes, including those in tumor nests. In tumor rich regions, tumor cell signal staining could bleed into the segmented immune cells and therefore there was no threshold for CK on immune cells. However, we did limit the threshold for CD8 and FoxP3 for a tumor cell. I have edited the text to clarify this point. We have clarified this on page 8 line 159:

The cells were classified as CD8+/FoxP3+ when the average intensity for both the CD8 and FoxP3 channel exceeded the threshold, CD8 when the average intensity of the CD8, but not the FoxP3, exceeded the threshold, and tumor only when the average intensity for the CK channel was above the threshold and below the threshold for FoxP3 and CD8.

8.) *With respect to dichotomizing various continuous variables (e.g., at the median value), while this historically has been common practice in translational and biomarker research, recent methodological guidance discourages this (refer the REMARK guideline paper linked above as well as https://doi.org/10.1136/bmj.332.7549.1080). I suggest the authors consider modelling continuous predictors and outcomes as continuous variables, including more contemporary techniques like restricted cubic splines to address non-linearity if encountered.*

AND

9.) *Relatedly and more seriously, using model results like hazard ratios to retrospectively merge quartiles is a practice at high risk of bias (see earlier links plus this more methodological resource https://doi.org/10.1002/sim.2331). I encourage the authors to reconsider this approach.*

**Reply:** As suggested, we now include analysis of the continuous spatial scores (page 12, line 266):

Continuous proximity, consistency and count scores were included in Cox proportional hazard models to assess the significance of the spatial measurements (Table 1). For interpretability, inverse hazard ratios are shown, so that higher values of continuous variables indicate a lower chance of recurrence. Count and proximity were normalized before inclusion in the model. Both adjusted and unadjusted hazards ratios were presented, with each spatial score adjusted for the other two variables. Interaction between proximity and count was also considered due to the moderate correlation between the two variables. All variables before adjusting showed evidence of association with recurrence, with the strongest evidence for proximity (p< 0.001) and consistency (p< 0.001). Proximity also had the largest unadjusted hazard ratio with a value of 2.46 (95% CI [1.54, 3.92]). After including all variables, proximity maintained the greatest effect size with a hazard ratio of 2.04 (95% CI [1.12, 3.70], p-value = 0.0189). Consistency showed some evidence of association with recurrence and had a hazard ratio of 1.14 (95% CI [1.00, 1.30], p-value = 0.0554). There was no evidence to suggest that count or the interaction between count and proximity were associated with recurrence after adjusting for other spatial metrics.

Table 1: Cox Proportional Hazards Model for Continuous Spatial Metrics

| **Covariates** | **Multivariate**  **HR (95% CI)** | **Multivariate p-value** | **Univariate**  **HR (95% CI)** | **Univariate**  **p-value** |
| --- | --- | --- | --- | --- |
| **Proximity** | 2.04 [1.12, 3.70] | 0.0189 | 2.46 [1.54, 3.92] | <0.001 |
|  |  |  |  |  |
| **Consistency** | 1.14 [1.00, 1.30] | 0.0554 | 1.23 [1.08, 1.37] | <0.001 |
|  |  |  |  |  |
| **Count**  **Proximity x Count** | 2.24 [0.65, 7.67]  1.62 [0.78, 3.36] | 0.1989  0.1965 | 1.24 [1.02, 1.51]  NA | 0.0341  NA |

10.) *When presenting results from a Cox regression, if a p-value is to be presented in addition the to the HR and CI then the p-value should come from the Cox model, not a log-rank test on the same data. The log-rank test can be used in a simpler, unadjusted analyses but mixing a log-rank test p-value with Cox regression results is confusing at best and misleading at worst.*

**Reply:** All p-values presented for the multivariate Cox proportional hazards models come from the CPH model. The log-rank test was only used when considering differences in between the Kaplan-Meier survival curve estimates. The methods section has been updated to clarify these methods (page 10, line 208):

The log-rank test was used to compute the p-values for difference between Kaplan-Meier estimates of survival curves. The hazard ratios and corresponding 95% confidence intervals are computed using the Cox proportional hazards model. All p-values for multivariate survival models come from the Cox proportional hazards model.

11.) *[MINOR] Relatedly, while hazard ratios are the generally accepted statistic for time-to-event analyses, they have some well-recognized limitations (see https://doi.org/10.1093/ehjacc/zuae017 and https://evidence.nejm.org/doi/10.1056/EVIDe2300142). The authors may wish to consider supplementing key HR outcomes with another statistic like difference in median survival or restricted mean survival time to assist with interpretability.*

**Reply:** We recognize the limitations of the hazard ratio, but felt that including other metrics would add too much length to the paper.

12.) *While the use of Spearman's rho as described is valid, it is limited in that it only provides an estimate of the association, not the effect size. Suggest the authors consider linear regression (or similar) either instead of or in addition to Spearman's rho, noting care must be taken when modelling a non-linear relationship.*

**Reply:** Methods such as linear regression are most useful in prediction problems. In this case it is not clear which variable is the predictor and the response, so correlation methods seem most appropriate.

13.) *The authors make many different references to "(statistical) significance". This is risky because the more claims of significance are made, the greater the risk of false positives and the more important it becomes to consider multiple testing correction strategies like the Bonferroni adjustment. Assuming the authors would prefer to avoid this, I'd suggest moderating or changing language away from statistical significance as an all-or-nothing threshold. One option is to talk in terms of strength of evidence e.g., "strong/moderate/some evidence of…" or "no evidence was found of…". I suggest avoiding statements like "borderline significantly" (particularly when the p > 0.05) and "… most significant association…" (for variable with smallest p-value in the model) as these can overinterpret and potentially mislead.*

**Reply:** As suggested, we have changed the wording in our text to refer to the strength of the evidence suggested by the p-values, for example on page 14, line 295:

Associations for count and proximity diverged when considering ER+ and ER- tumors together. Among breast cancers overall, high proximity and high consistency both showed strong evidence of association with recurrence (p-values < 0.001), while there was little evidence that lymphocyte count was associated with recurrence (Fig 4, top row).

14.) *There are many methods discussed for the first time in the Results. Suggest ensuring the demarcation between Methods and Results is carefully observed.*

**Reply:** We have made changes to ensure that all methods are first covered in the Methods section, with only brief reiteration in results for clarity.

15*.) Similarly, suggest ensuring any non-trivial interpretation of Results is presented in the Discussion, not the Results.*

**Reply:** We have removed interpretations from the results section.

16.) *[MINOR] Avoid using exponential notation - for p-values suggest something like "p<.0001" (or similar) for very small values.*

**Reply:** The text no longer has p-values expressed in scientific notation. We have not changed the p-values in tables and figures because they reflect relevant differences in strengths of the evidence as discussed in the text.

17.) *I found Figure 4 quite difficult to understand - perhaps revisit and ensure spacing and labelling are correct.*

**Reply:** We have edited the figure and legend for clarity and to ensure consistent spacing and reduce confusion.

18*.) I understand and agree with the authors' use of the LRT to assess the joint contribution of proximity and consistency to the Cox proportional hazards model. That said, care must be taken when interpreting the results. A small p-value indicates that the variables improve model fit but does not necessarily mean this improvement is clinically meaningful or that the added variables provide substantial predictive value.*

**Reply:** We agree with this important principle of hypothesis testing and ensured that the text does not imply that improved model fit guarantees that a variable is clinically meaningful.

19.) *I have already noted that much of the Results are actually methods that should be moved to the Methods section. I would also suggest more detail be added to the Methods about the modelling underpinning the analyses in Table 1, because currently odds ratios are presented without clearly specifying the generating model.*

**Reply:** We added details to the methods to clarify the generating model used when computing the odds ratios on page 10, line 218, as follows:

Odds ratios and 95% confidence intervals were computed assuming the data was generated by a multinomial model to determine the level of association of proximity, consistency, and lymphocyte count with AGI classified participants and to determine the level of association of proximity, consistency, and lymphocyte count with Adaptive-Enriched participants.

20.) *Notwithstanding my earlier comment about ensuring interpretation is moved out of the Methods into the Discussion, I found the current Discussion was clear, contextualised the results well, and effectively linked results to potential clinical impacts. I had just one query: the authors indicated some TMA cores were lost because of QC check failures. Is this a limitation? Is there any risk the loss of these TMA cores could introduce bias?*

**Reply:** To assess the reviewers important point, we evaluated drop out for changes in the distribution of demographic or clinical variables and have revised the text to reflect that drop out was not associated with clinical variables (page 6, line 111):

Distributions of common demographic and clinical variables were computed before and after quality control to ensure that reduction in participants did not introduce bias (Fig 1). We did not observe any substantial changes in the distribution of these variables.

Reviewer #2

1.) *Early-stage breast cancers will have different morphologic features and cellular dispersion patterns, for example, ILC can grow in linear cell bundles, low grade have well defined epithelial nests that often exclude TILs, high grade tumors can be more dispersed/confluent with loss of ductal architecture. I worry that the spatial pattern of the tumor cells will alter the tumor-CD8 NND, and that the spatial pattern of the tumor cells will be correlated with other prognostic features such as tumor grade and subtype, and that this raises the risk of confounding. It would be important to address this in the data somehow. Could you evaluate the spatial patterns of CK+ clustering and see how it correlates with your metric, and see if it influences outcomes of your prediction models.*

AND

2.) *In relation to comment 1- please evaluate clinical prognostic factors from the cohort that could be added to the multivariate models, to see if the spatial metrics maintain significance. Grade, tumor size/nodal status, ILC v. IDC, age/menopause status, etc. Look for interaction effects to see if there are differences in prognostic impact according to the covariates.*

**Reply:** To address the concern of potential confounding by other prognostic features, we have included results from a Cox proportional hazards model that includes common clinical variables such as age, grade, stage, ER status, PR status, and HER2 status. After adjusting, proximity remains significant and has strong magnitude, as revised on page 13, line 281:

Proximity and consistency were included in a Cox proportional hazards model with important clinical and demographic variables (Table 2). The variables we adjusted for were age, stage, grade, ER status, PR status, and HER2 status. Count was excluded because it showed the weakest evidence of association in the previous model. After adjusting, proximity continues to show strong evidence of association with a hazard ratio of 2.88( 95% CI [1.70, 4.90], p-value <0.0001). This is also the largest hazard ratio of all variables included in the model. However, there is no evidence that consistency is associated with recurrence after adjusting for clinical variables and age (p-value = 0.2060).

3.) *On a similar note, in early-stage breast cancer we have usually have distinct stromal and intraepithelial tissue compartments, and the TILs behave differently across the two compartments, i.e. TILs usually found in stroma but less so in the intraepithelial nests. would your NND metric be influenced unduly in this case by variations in stromal/intraepithelial sampling across the TMAs? You can explore this by quantifying the relative degree of stroma v. epithelia in each sample, and see if this changes the readout.*

**Reply:** Our IF stained TMA cores do not have a marker for stroma and we were unable to differentiate intratumoral and stromal lymphocytes with our methods. We have clarified in the text that proximity is, by definition, a measure of the distance from tumor cells to CD8 T cells irrespective of compartment. We also contrast our method with those of other investigators who distinguished stromal and intratumoral infiltrates (page 19, line 421):

Our work is consistent with several previous studies demonstrating that higher levels of CD8+ T-cells are associated with positive prognosis in TNBC[29,30]. Most of these studies have suggested limited or no benefit of CD8+ infiltration for ER+ participants[31,32]. However, one prior study documented CD8 T-cells in stroma and intratumoral regions and established an association between intratumoral T-cells and survival for both ER+/HER2- and ER- participants[33].

 4.) *NND and immune cell density can also be influenced by the location of the spatial sample in relation to tumor invasive margin/front. Was this accounted for when the TMA regions were selected? Please describe how in the manuscript.*

**Reply:** All TMA cores were taken from intratumoral regions identified by pathologists as epithelial rich. We have included these details in the methods (page 7, line 146):

Multiplex IF images consisted of slides of multiple 1mm TMA cores, where most participants had up to four cores and a small group of participants, who had two tissue samples taken, had up to eight cores. TMA cores were taken from a pathologist-identified intratumoral region selected for a high percentage of epithelium.

5.) *If you have substantial inter-tumoral heterogeneity you may not accurately capture the mean for the parent specimen from the TMA. It would be interesting to evaluate heterogeneity of your metrics across cores from the same patient. Can you compare the accuracy of your metrics from TMA versus WSI to see if you are getting enough sample? Does increasing # of TMAs improve the accuracy of your metrics in comparison to the WSI?*

**Reply:** One of the purposes of our consistency metric was to capture cross-core heterogeneity. For example, if one core had many CD8 T cells close to tumor cells and another had CD8 T cells far from tumor cells, then the tumor may have moderate to  high proximity, but lower consistency. Proximity can be computed at the core level, but interpretation of consistency is not very interpretable as a per core measurement. We have modified the text to emphasize this point on page 8 line 177:

The collection of log10 Nearest Neighbor Distances (NNDs) for each TMA core corresponding to a single study ID were aggregated and the mean and variance for the distribution of aggregated log10 NNDs was computed for each study ID. The consistency metric was interpreted as a measure of intra-individual and cross-core heterogeneity.

6*.) As you mention in the discussion and elsewhere, CD8/CK NND and CD8 density may interact, i.e. different association of NND with outcome across different CD8 density levels. Have you explored CPH models with interaction terms for the two predictor variables?*

**Reply:** As suggested, we have included Cox proportional hazards models that consider interaction between count and proximity. The results are given in Table 1 and summarized on page 12, line 274:

In multivariate models adjusted for other spatial factors, proximity maintained a strong effect size with a hazard ratio of 2.04 (95% CI [1.12, 3.70], p-value = 0.0189). Consistency showed some evidence of association with recurrence and had a hazard ratio of 1.14 (95% CI [1.00, 1.30], p-value = 0.0554) for RFS overall. There was no evidence to suggest that count or the interaction between count and proximity were associated with recurrence after adjusting for other spatial metrics.

7.) Density plots in fig 2 are a little confusing to interpret because the cumulative density is split across the subgroups. Could you instead overlay the densities so that for each subgroup the cumulative density is =1, that way you can see how each subgroup differs in terms of their spatial patterns?

**Reply:** Visual insight into probability distributions come from areas under the density curve. In the case of subgroups, that intuition is enhanced by making the sum of the sum of the subgroup areas equal to 1. This is especially meaningful when the subgroups have different sizes as in our dataset. Making each subgroup density equal to 1 loses the important information about differing subgroup sizes.

8.) *What about associations of T reg proximity with outcome? T-regs may interfere with CD8s- could you look at associations of CD8-Treg NND with your other metrics, and with outcome?*

**Reply:** T regulatory cells are classically CD4+/FoxP3+ and CD8-. We have edited the text to remove mention of Treg cells and to describe the excluded cell population as CD8+/FoxP3+. We excluded FoxP3+ cells due to high variability in the FoxP3 staining, however we have now added sensitivity analyses with and without exclusion of these cell types (supplemental figure 1).

9.) *What is the path forward here for taking this to this clinic? mIF very expensive and not clinically validated. You mention H&E ML TILs classifiers in the discussion. can you analyze your H&E WSI images metrics using these classifiers? Can you look at the same metrics just using H&E?*

**Reply:** We included WSI metrics to facilitate biological interpretation of our spatial metrics. While proximity and consistency could be applied to H&E-stained tissues, we did not pursue this as a first step. We have addressed this on page 21, line 471:

 Multiplex IF staining is increasingly cost-effective and efficient, allowing for viable implementation in clinical settings[19]. Therefore, future clinical studies may be able to use proximity and consistency of multiplex IF-derived spatial immune markers to predict response to immunotherapy. It is also possible that H&E-derived distance metrics, if validated, could be useful. Given that ER+ tumors represent the majority of breast tumors, closer evaluation of immune response to identify possible beneficiaries is highly impactful.

Reviewer #3

1.) *Please review the REMARK checklist for tumor biomarker studies and include any missing components to improve transparency. For instance, it is unclear whether spatial measurements were conducted blinded to outcome data.*

**Reply:** The spatial measurements were conducted prior to receiving clinical data. We have made necessary changes to the methods to clarify the point and the revised text is included below. Furthermore, we have reviewed the REMARK checklist and ensured that all other necessary components have been included as noted on page 8, line 173:

The spatial biomarker values were computed in Python and all spatial measurements were conducted prior to receiving clinical data. The Nearest Neighbor Distance (NND) from a tumor cell to the CD8 T cell was computed for each TMA core.

2.) *The manuscript occasionally shifts between present and past tense in sections such as Image Processing. Consider reviewing and standardizing the tense throughout, ideally using the past tense to describe methods and results.*

**Reply:** The inconsistencies in tense have been addressed and any cases of present tense in the methods and results have been changed to past tense.

3.) *The authors define regulatory T cells as CD8⁺FOXP3⁺, but regulatory T cells are classically CD4⁺FOXP3⁺ and CD8⁻. Figure 1 appears to show many cells labeled as Tregs that express CD8, which would be highly unusual. Could this reflect fluorescence signal bleed-through or misclassification? Have the authors evaluated marker expression within distinct cellular compartments (e.g., nuclear vs. membrane localization)?*

**Reply:** T regulatory cells are classically CD4+/FoxP3+ and CD8-. We have edited the text to remove mention of Treg cells and to describe the excluded cell population as CD8+/FoxP3+. We excluded FoxP3+ cells due to high variability in the FoxP3 staining, however we have now added sensitivity analyses with and without exclusion of these cell types (supplemental figure 1).

4.) *How was the multiplex immunofluorescence assay validated? The authors are encouraged to review best practices, such as those outlined in the SITC statement (PMID: 32414858), and confirm that staining patterns in the multiplex assay reflect those observed in single-plex IHC.*

Reply: To confirm the quantitation of multiplex assay results based on Qupath, we calculated associations between QuPath lymphocyte counts and Halo-derived estimates on the same IF. To confirm the biological validity of the staining, we evaluated associations between CD8 protein expression and CD8A RNA levels in the same specimens (although different cores). We report these methodological details in Methods on page 8, line 163:

QuPath quantitation of CD8 was highly correlated with Halo quantitation on the same slides (rho = 0.97). QuPath CD8 density measures (counts per unit area) were also correlated with RNA expression data from separate cores from the same tumor specimen (rho = 0.49).

5.) *Were lymphocyte counts normalized to the tissue area analyzed? Omitting this step may reduce the accuracy and prognostic relevance of lymphocyte count.*

**Reply:** All the cores in our TMA were 1mm punches and therefore variation would be related to stromal or adipose related dropout. To standardize to tumoral area irrespective of content, we did not adjust for these differences. Moreover, when aggregating cell counts at the participant level, we used the average lymphocyte count across all TMA cores to address variation in core content and has been noted on page 9, line 190:

The lymphocyte count was computed as the total number of cells which were classified as CD8 T cells and CD8+/FoxP3+ cells. Lymphocyte count at the participant level is computed as the average of lymphocyte counts over all of the participants cores.

6.) *Please provide version numbers for all R packages used in the statistical analyses to ensure reproducibility.*

**Reply:** The version numbers for R packages used in statistical analysis have been included as well as names and version numbers of Python packages used (page 10, line 222):

All statistical analysis was performed using SciPy version 1.14.1 in python except survival analysis. Kaplan-Meier curves were computed using Lifelines version 0.29.0 in python and the Cox proportional hazards models were computed using Survival 3.5.5 in R.

7.) *Because proximity to the nearest lymphocyte is inherently influenced by overall lymphocyte density, a formal correlation analysis between proximity, consistency, and total lymphocyte count would strengthen the results and should be reported and discussed.*

**Reply:** We have revised the results to discuss the correlation between spatial metrics (page 12, line 260):

Finally, we considered the levels of correlation between all computed spatial metrics (Supplemental Table 7 in S1 Supplemental Materials). Proximity and count showed moderate levels of correlation (corr. = 0.5545). This was not unexpected, given that cell distances are inherently influenced by cell density. However, the correlation between count and consistency was low (corr. = 0.3261). Furthermore, proximity and consistency had a relatively low correlation (corr. = 0.4717).

8.) *Consider changing the X-axis of Kaplan-Meier curves from days to years for interpretability and including number-at-risk tables below each curve.*

**Reply:** As suggested, we have changed the time from days to years on the x-axis of Kaplan-Meier curves. We have decided not to include number-at-risk tables so as not to overcrowd the figures.

9*.) Were multivariable Cox regression models performed to adjust for conventional prognostic factors (e.g., tumor size, nodal status, grade)? If not, this should be considered to demonstrate the independent prognostic value of the spatial metrics.*

**Reply:** To address the concern of potential confounding by other prognostic features, we have included results from a Cox proportional hazards model that includes common clinical variables such as age, grade, stage, ER status, PR status, and HER2 status. After adjusting, proximity remains significant and has strong magnitude, as revised on page 13, line 281:

Proximity and consistency were included in a Cox proportional hazards model with important clinical and demographic variables (Table 2). The variables we adjusted for were age, stage, grade, ER status, PR status, and HER2 status. Count was excluded because it showed the weakest evidence of association in the previous model. After adjusting, proximity continues to show strong evidence of association with a hazard ratio of 2.88( 95% CI [1.70, 4.90], p-value <0.0001). This is also the largest hazard ratio of all variables included in the model. However, there is no evidence that consistency is associated with recurrence after adjusting for clinical variables and age (p-value = 0.2060).

10.) *Given that regulatory T cells were included in the staining panel, why were no spatial or density-based analyses of Tregs reported? Including such analyses could provide additional insights.*

**Reply:** T regulatory cells are classically CD4+/FoxP3+ and CD8-. We have edited the text to remove mention of Treg cells and to describe the excluded cell population as CD8+/FoxP3+. We excluded FoxP3+ cells due to high variability in the FoxP3 staining, however we have now added sensitivity analyses with and without exclusion of these cell types (supplemental figure 1).

Reviewer #4

Weaknesses and Limitations

1.) *Only CD8+ and FoxP3+ cells are considered. The exclusion of CD4+ helper T cells, B cells, macrophages, and other immune subsets may oversimplify the TIME.*

**Reply:** Our TMA cores were only stained for CD8 and FoxP3 markers. In future analysis, we hope to include other immune cell markers.

2.) *The metric for Consistency (variance of log-transformed distances) is less intuitive and harder to interpret biologically. Its weaker and less consistent statistical significance also raises concerns about robustness.*

**Reply:** While we agree that consistency is not as statistically robust as proximity, it does provide complementary information that could help determine the effectiveness of immune infiltration, especially when lymphocyte count is relatively high.

3.) *TMAs cover only a small tumor area (1 mm cores), potentially missing heterogeneity present in full tumor sections. While the authors compare TMA and WSI metrics, validation in WSI settings would strengthen generalizability.*

**Reply:** We acknowledge this is a limitation of our study and included this in the discussion (page 20, line 463 given below). However, the immunofluorescent staining was not performed at the whole slide level and obtaining IF stained WSI data is cost prohibitive for our cohort of over 1700 women. The TMA includes multiple cores per patient from across the whole slide image allowing us to capture the heterogeneity present in the tumor. Though we are capturing small regions of the tumor, proximity and consistency were linked to spatial scores measured at the whole slide image level as stated on page 16, line 361:

Proximity, consistency, and lymphocyte count were all correlated with the four WSI TIL scores (Figure 6). We observed the strongest correlations between proximity and intratumoral strength (corr. = 0.598), but also observed associations of proximity with pertumoral strength (corr. = 0.567) and TIL forests (corr. = 0.503) and an inverse association with TIL deserts (corr. = -0.469) (Figure 6).

A limitation of our analysis is that we did not validate our IF measures across the larger area of whole slides, however, we find it promising that proximity and consistency were associated with whole slide immune features.

4.) *Supplementary Table 3 shows clear signs of overfitting in training/testing splits when optimizing cut-points for biomarkers, reducing confidence in dichotomization strategies.*

**Reply:** We clarified how we established cutpoints and also show a sensitivity analysis where we optimized cutpoints in a 2/3 training and 1/3 test set (Supplemental Table 3 and description on page 9, line 193):

When participants were split based on high/low lymphocyte count, the median count was used as the cut-point. Proximity and consistency were binarized for the purpose of comparing differences in recurrence between participants with high vs. low values of proximity/consistency. We explored various cutpoints for proximity and consistency. The cutpoint used in the analysis was based on splitting the data into 4 groups based on the quartiles and then collapsing groups which had similar survival curves. After collapsing groups, the median was the final cutpoint for consistency and the 1^st^ quartile was the final cutpoint for proximity.

5.) *While CBCS data is available upon request, the requirement for a data use agreement limits replicability and open access principles.*

**Reply:** Due to the data use agreements for CBCS, the data is not publicly available. However, investigators may submit a letter of intent to gain access upon reasonable request and IRB approval.

6.) *Findings are derived from a single cohort. Independent validation in external datasets or clinical trials would enhance credibility.*

**Reply:** We did not have access to an external dataset of IF stained TMA cores. We acknowledge this is a limitation. As always, validation of the results observed would be valuable, however, optimization of similar calculations form H&E would be more likely to result in comparable cohorts as few datasets have similar IF panels.

7.) *Assumes that all CD8+ cells are functional cytotoxic T cells, which may not always be the case (e.g., exhausted or anergic phenotypes are not considered).*

**Reply:** The functional status of cytotoxic T cells was not available for this analysis.

8.) *While the implications for immunotherapy are mentioned, the manuscript could further elaborate on how these spatial biomarkers might be integrated into clinical workflows or guide treatment decisions.*

**Reply:** We have edited the text to expand on how spatial biomarkers might be included in a clinical setting on page 21, line 471:

Multiplex IF staining is increasingly cost-effective and efficient, allowing for viable implementation in clinical settings[19]. Therefore, future clinical studies may be able to use proximity and consistency of multiplex IF-derived spatial immune markers to predict response to immunotherapy. It is also possible that H&E-derived distance metrics, if validated, could be useful. Given that ER+ tumors represent the majority of breast tumors, closer evaluation of immune response to identify possible beneficiaries is highly impactful.

Suggestions for Improvement

1.) *Add multivariate models controlling for clinical covariates (e.g., age, tumor grade, nodal status) to strengthen claims of independent prognostic value.*

**Reply:** To address the concern of potential confounding by other prognostic features, we have included results from a Cox proportional hazards model that includes common clinical variables such as age, grade, stage, ER status, PR status, and HER2 status. After adjusting, proximity remains significant and has strong magnitude, as revised on page 13, line 281:

Proximity and consistency were included in a Cox proportional hazards model with important clinical and demographic variables (Table 2). The variables we adjusted for were age, stage, grade, ER status, PR status, and HER2 status. Count was excluded because it showed the weakest evidence of association in the previous model. After adjusting, proximity continues to show strong evidence of association with a hazard ratio of 2.88( 95% CI [1.70, 4.90], p-value <0.0001). This is also the largest hazard ratio of all variables included in the model. However, there is no evidence that consistency is associated with recurrence after adjusting for clinical variables and age (p-value = 0.2060).

2.) *Clarify consistency metric's biological rationale—possibly explore alternative metrics (e.g., Gini index, spatial autocorrelation).*

**Reply:** Consistency is a metric of variation in distances in relation to the tumor cells as opposed to spatial autocorrelation which is related to cell dispersion within the core. We have revised the text to clarify the biological rationale of consistency (page 18, line 413).

Furthermore, Consistency measures the level of uniformity of CD8 T cells among tumor cells as well as the inter-core heterogeneity of immune response. A more uniform immune response may imply greater efficiency in the cytotoxic effects of CD8 T cells.

3.) *Consider evaluating CD8+ functional status (e.g., granzyme B, PD-1 expression) in future studies.*

**Reply:** We agree and hope to consider functional status in future studies.

4.) *Include visual examples or distributions of spatial metrics in different subtypes to better illustrate spatial heterogeneity.*

**Reply:** We have edited Figure 2 to include an example from a TMA core that would have low proximity. Furthermore, Figure 3 in the manuscript shows kernel density estimates of the distributions of count, proximity and consistency with sub-density plots for ER status and PAM50 subtype.

5.) *Expand on translational relevance—e.g., could spatial metrics predict immunotherapy response in ER+ subtypes?*

**Reply:** We have edited the text to expand on how spatial biomarkers might be included in a clinical setting on page 21, line 471:

Multiplex IF staining is increasingly cost-effective and efficient, allowing for viable implementation in clinical settings[19]. Therefore, future clinical studies may be able to use proximity and consistency of multiplex IF-derived spatial immune markers to predict response to immunotherapy. It is also possible that H&E-derived distance metrics, if validated, could be useful. Given that ER+ tumors represent the majority of breast tumors, closer evaluation of immune response to identify possible beneficiaries is highly impactful.

Specific Editorial Comments

1) *Please ensure that the funders and grant numbers match between the Financial Disclosure field and the Funding Information tab in your submission form. Note that the funders must be provided in the same order in both places as well. State what role the funders took in the study. If the funders had no role in your study, please state: “The funders had no role in study design, data collection and analysis, decision to publish, or preparation of the manuscript.”*

**Reply:** We have made these changes to funder information upon resubmission.

2) *Please provide separate figure files in .tif or .eps format.*

**Reply:** Figures have been included as separate .tif files and removed from manuscript. Figure names and titles have been place in the manuscript following the paragraph they are first mentioned.

3) *We notice that your supplementary Figures and Tables are included in the manuscript file. Please remove them and upload them with the file type 'Supporting Information'. Please ensure that each Supporting Information file has a legend listed in the manuscript after the references list.*

**Reply:** All supporting information has been moved to a file called S1_Supplemental_Materials. We have also included the legend after the references.

4*) We do not publish any copyright or trademark symbols that usually accompany proprietary names, eg ©, ®, ™ (e.g. next to drug or reagent names). Please remove all instances of trademark/copyright symbols throughout the text, including: ™ on page 5.*

**Reply:** We have removed the trademark symbol and ensured that there are no other copyright or trademark symbols included.

General Editorial Comments

1.) *We ask every co-author listed on the manuscript to fill in a contributing author statement, making sure to declare all competing interests. If any of the co-authors have not filled in the statement, we will remind them to do so when the paper is revised. If all statements are not completed in a timely fashion this could hold up the re-review process. If new competing interests are declared later in the revision process, this may also hold up the submission. Should there be a problem getting one of your co-authors to fill in a statement we will be in contact. Please do not add or remove authors without first discussing this with the handling editor. You can see our competing interests policy here:* [*http://journals.plos.org/plosmedicine/s/competing-interests*](http://journals.plos.org/plosmedicine/s/competing-interests)*.*

**Reply:** All authors are aware that they must fill out a contributing author statement.

2.)  *Please upload any figures associated with your paper as individual TIF or EPS files with 300dpi resolution at resubmission; please read our figure guidelines for more information on our requirements: http://journals.plos.org/plosmedicine/s/figures. While revising your submission, please upload your figure files to the PACE digital diagnostic tool, https://pacev2.apexcovantage.com/. PACE helps ensure that figures meet PLOS requirements. To use PACE, you must first register as a user. Then, login and navigate to the UPLOAD tab, where you will find detailed instructions on how to use the tool. If you encounter any issues or have any questions when using PACE, please email us at* [*PLOSMedicine@plos.org*](mailto:PLOSMedicine@plos.org)*.*

**Reply:** This comment has already been addressed in Specific Editorial Comments (2).

3.)  *Please ensure that the paper adheres to the PLOS Data Availability Policy (see http://journals.plos.org/plosmedicine/s/data-availability), which requires that all data underlying the study's findings be provided in a repository or as Supporting Information. For data residing with a third party, authors are required to provide instructions with contact information (web or email address) for obtaining the data. Please note that a study author cannot be the contact person for the data. PLOS journals do not allow statements supported by "data not shown" or "unpublished results." For such statements, authors must provide supporting data or cite public sources that include it.*

**Reply:** Our paper adheres to the PLOS Data Availability Policy. In our case, due to the CBCS data sharing agreement, we cannot make data publicly available. However, CBCS data are available upon request (<https://unclineberger.org/cbcs>). This has been made clear in the Data Availability section of out manuscript.

4*.) We expect all researchers with submissions to PLOS in which author-generated code underpins the findings in the manuscript to make all author-generated code available without restrictions upon publication of the work. In cases where code is central to the manuscript, we may require the code to be made available as a condition of publication. Authors are responsible for ensuring that the code is reusable and well documented. Please make any custom code available, either as part of your data deposition or as a supplementary file. Please add a sentence to your data availability statement regarding any code used in the study, e.g. "The code used in the analysis is available from Github [URL] and archived in Zenodo [DOI link]" Please review our guidelines at https://journals.plos.org/plosmedicine/s/materials-software-and-code-sharing and ensure that your code is shared in a way that follows best practice and facilitates reproducibility and reuse. Because Github depositions can be readily changed or deleted, we encourage you to make a permanent DOI'd copy (e.g. in Zenodo) and provide the URL.*

**Reply:** All image analysis was performed in QuPath, an open source image analysis and digital pathology software, using built in functionality and all statistical and spatial analysis was performed in Python or R using well established methods such as Nearest Neighbor Distance and Cox proportional hazards models that are available through publicly available packages.

5.)  *FINANCIAL DISCLOSURES: The funding statement should include: specific grant numbers, initials of authors who received each award, URLs to sponsors’ websites. Also, please state whether any sponsors or funders (other than the named authors) played any role in study design, data collection and analysis, the decision to publish, or preparation of the manuscript. If they had no role in the research, include this sentence: “The funders had no role in study design, data collection and analysis, decision to publish, or preparation of the manuscript.”*

**Reply:** We addressed this issue in Specific Editorial Comments (1)

6*.)  In the manuscript text, please indicate: (1) the specific hypotheses you intended to test, (2) the analytical methods by which you planned to test them, (3) the analyses you actually performed, and (4) when reported analyses differ from those that were planned, transparent explanations for differences that affect the reliability of the study's results. If a reported analysis was performed based on an interesting but unanticipated pattern in the data, please be clear that the analysis was data driven.*

**Reply:** All components have been included.

7.) *Please state in the Methods section whether the study had a prospective protocol or analysis plan. If a prospective analysis plan (from your funding proposal, IRB or other ethics committee submission, study protocol, or other planning document written before analyzing the data) was used in designing the study, please include the relevant document(s) with your revised manuscript as a Supporting Information file to be published alongside your study and cite it in the Methods section. A legend for this file should be included at the end of your manuscript. If no such document exists, please make sure that the Methods section transparently describes when analyses were planned, and when/why any data-driven changes to analyses took place. Changes in the analysis, including those made in response to peer review comments, should be identified as such in the Methods section of the paper, with rationale.*

**Reply:** Our study did not have a prospective analysis plan. All details have been appropriately described in the Methods section.

8.) *Abstract: Please structure your abstract using the PLOS Medicine headings (Background, Methods and Findings, Conclusions). Please combine the Methods and Findings sections into one section.*

**Reply:** The abstract has been restructured using the PLOS Medicine headings.

9.)  *At this stage, we ask that you include a short, non-technical Author Summary of your research to make findings accessible to a wide audience that includes both scientists and non-scientists. The Author Summary should immediately follow the Abstract in your revised manuscript. This text is subject to editorial change and should be distinct from the scientific abstract. Ideally each sub-heading should contain 2-3 single sentence, concise bullet points containing the most salient points from your study. In the final bullet point of 'What Do These Findings Mean?', please include the main limitations of the study in non-technical language. Please see our author guidelines for more information: https://journals.plos.org/plosmedicine/s/revising-your-manuscript#loc-author-summary.*

**Reply:** An Author Summary has been included in the manuscript following the abstract.

10.)  *Please express the main results with 95% CIs as well as p values. When reporting p values please report as p<0.001 and where higher as the exact p value p=0.002, for example. Throughout, suggest reporting statistical information as follows to improve clarity for the reader "22% (95% CI [13%,28%]; p</=)". Please be sure to define all numerical values at first use.*

**Reply:** 95% confidence intervals and p-values are presented for main results. P-values have been edited to report as specified.

11.) *Please include page numbers and line numbers in the manuscript file. Use continuous line numbers (do not restart the numbering on each page).*

**Reply:** Page numbers and continuous line numbers have been added to the manuscript.

12.) *Please cite the reference numbers in square brackets. Citations should precede punctuation.*

**Reply:** The manuscript has been edited to ensure in-text citations are placed in brackets and precede punctuation.

13.)  *Please provide titles and legends for all figures and tables (including those in Supporting Information files).*

**Reply:** Titles and legends have been provided for all figures. Tables have been provided with titles.

14.)  *Please define all abbreviations used in each figure/table (including those in Supporting Information files).*

**Reply:** All abbreviations used in figures and tables have been defined.

15.)  *Please consider avoiding the use of red and green in order to make your figure more accessible to those with color blindness.*

**Reply:** The use of green and red in figures have been avoided as much as possible, however, given that some of the biomarkers were stained green, this could not be entirely avoided.

16.)  *Please note that supplementary material will be posted as supplied by the authors. Therefore, please amend it according to the relevant comments outlined here.*

**Reply:** The presentation of the supplementary materials is satisfactory to the authors.

17.) *Please cite your Supporting Information as outlined here: https://journals.plos.org/plosmedicine/s/supporting-information*

**Reply:** In-text citations of supporting materials have been edited to conform to the outlined format.

18.) *PLOS uses the numbered citation (citation-sequence) method and first six authors, et al.*

**Reply:** citations have been edited to use the Vancouver style with the above method for listing authors.

19.) *Please ensure that journal name abbreviations match those found in the National Center for Biotechnology Information (NCBI) databases (http://www.ncbi.nlm.nih.gov/nlmcatalog/journals), and are appropriately formatted and capitalised.*

**Reply:** To the best of our knowledge, appropriate abbreviations, formatting, and capitalization have been used for journal names.

20.)  *Where website addresses are cited, please include the complete URL and specify the date of access (e.g. [accessed: 12/06/2023]).*

**Reply:** Where relevant, URL’s and date of access has been included.
