# Supplementary material for: Prognostic significance of CD8+ T cell Spatial Biomarkers in ER+ and ER− breast cancer: A retrospective cohort study
Source: PLoS Med. 2025 Oct 15;22(10):e1004647. doi: 10.1371/journal.pmed.1004647 (PMC12539700; doi:10.1371/journal.pmed.1004647)
Supplement: Attachment — Submitted filename: Response_to_Reviewer_Comments_PMEDICINE-D-25-01829R2_AEW.docx [file pmed.1004647.s014.docx]

We would like to thank the reviewers and editor for their time, as well as the opportunity to revise our manuscript. Reviewer comments are listed in *italics* with our responses below each comment. Responses and text excerpts are given in blue.

Reviewer #1

10.) *My understanding is the authors agree with my suggestion not to combine hazard ratios with log-rank test p-values, however this is still present in the abstract "(HR 1.35, 95% CI [0.92,1.98], log-rank p-value = 0.13)". Again, I suggest only presenting Cox model p-values with hazard ratios.*

**Reply:** We have removed the log-rank p-value from the abstract and replaced with the p-value from the CPH model.

12.) *I am afraid I must disagree with the authors on this point. Linear regression absolutely can be used for investigating associative relationships and is not just for "prediction problems" per the authors' response to my review comment. I take the authors' point that the assignment of "outcome" and "covariate" to the two variables of interest can be arbitrary in some cases, but this does not obviate my point that rho only estimates association whereas linear regression provides a meaningful estimate of effect.*

**Reply:** We agree with the reviewer and did not intend to imply that linear regression is only useful in prediction problems. Linear regression can be used to investigate associative relationships and also provides an estimate of the relative change in a response variable with respect to a covariate of interest. When comparing these two different methods of measuring tumor infiltration, we are only interested in the association ( i.e. how well correlated) the two scores are. Below we have provided the results from linear regression between our computed spatial metrics (count, proximity, and consistency) as the predictor and each of the whole slide image scores (Intratumoral Strength, Peritumoral strength, Intratumoral desert, and Intratumoral forest) as the response variables. We have chosen not to include this in the paper because we feel that it does not add much beyond what was already given by the first correlation analysis (using spearman’s rho).

| Predictor  (Spatial Metrics  From TMA) | | Response (Whole Slide Image Scores) | | | |
| --- | --- | --- | --- | --- | --- |
|  |  | Intratumoral  Strength | Peritumoral  Strength | Intratumoral  Desert | Intratumoral  Forest |
| Log10(count) | Slope  (95% CI) | 0.57 (0.44,0.69) | 0.69 (0.54,0.84) | -0.27  (-0.37,-0.18) | 0.36  (0.27,0.45) |
|  | Intercept  (95% CI) | -0.14  (-0.46,0.18) | 0.13  (-0.26,0.51) | 1.26 (1.01,1.51) | -0.53  (-0.77,-0.30) |
|  | Pearson r | 0.54 | 0.55 | -0.37 | 0.49 |
| proximity | Slope  (95% CI) | 1.32 (1.08,1.55) | 1.27 (0.96,1.59) | -0.70  (-0.89,-0.51) | 0.75  (0.57,0.93) |
|  | Intercept  (95% CI) | -0.25  (-0.53,0.04) | 0.38 (0.00,0.77) | 1.39 (1.16,1.62) | -0.50  (-0.72,-0.28) |
|  | Pearson r | 0.62 | 0.49 | -0.46 | 0.50 |
| consistency | Slope  (95% CI) | 7.85 (5.11,10.60) | 7.07 (3.64,10.49) | -5.01  (-7.01,-3.02) | 4.63  (2.66,6.61) |
|  | Intercept  (95% CI) | -5.80  (-8.29,-3.31) | -4.51  (-7.61,-1.41) | 5.09 (3.29,6.90) | -3.81  (-5.59,-2.02) |
|  | Pearson r | 0.36 | 0.27 | -0.32 | 0.30 |

Editorial Comments: General

1.)  *Please confirm that your title complies with to PLOS Medicine's style. Your title must be nondeclarative and not a question. It should begin with main concept if possible. "Effect of" should be used only if causality can be inferred, i.e., for an RCT. Please place the study design ("A randomized controlled trial," "A retrospective study," "A modelling study," etc.) in the subtitle (ie, after a colon).*

**Reply:** We have revised the title to include “A Retrospective Cohort Study” as the subtitle.

2.)  *Statistical reporting: Please revise throughout the manuscript, including tables and figures.
- Please report statistical information as follows to improve clarity for the reader ""22% (95% CI [13,28]; p</=)"".
- Please separate upper and lower bounds with commas instead of hyphens as the latter can be confused with reporting of negative values.
- Please repeat statistical definitions (HR, CI etc.) for each set of parentheses.*

**Reply:** We have edited the statistical reporting to include p-values in addition to the confidence intervals and formatted the information as suggested. Where statistical definitions were not repeated within the parentheses, this has been corrected. All confidence intervals are reported with commas between lower and upper bounds.

3.)  *Please ensure that all abbreviations are defined at first use throughout the text (including statistical abbreviations).*

**Reply:** We have made changes to ensure that analysis of variance (ANOVA) and Odds Ratio (OR) are defined before abbreviations are used. All other abbreviations should already be defined before first use.

4.)  *Please ensure that tables and figures, including those in supplementary files, are appropriately referenced in the main text.*

**Reply:** We have checked to ensure all tables and figure are appropriately referenced and made changes where necessary.

5.) *Please review your text for claims of novelty or primacy (e.g. 'for the first time' or ‘novel’) and remove this language.*

**Reply:** We have removed any language implying novelty or primacy.

6.) *Please confirm that any use of statistical terms (such as trend or significant) are supported by the data, and if not please remove them. The term trend should be used only when the test for trend has been conducted.*

**Reply:** All uses of the term “significant” are supported by the results from the data. The word “trend” is not used in the paper.

7.)  *Please provide titles and legends for all figures and tables (including those in Supporting Information files).*

**Reply:** We have ensured that all figures and tables include a titles, including supplementary figures and tables that did not previously have a title. Most figures include a legend, but not all. PLOS figure guidelines state that legends are optional.

8.) *Please define all acronyms used in each figure or table in its corresponding legend.*

**Reply:** As requested we have ensured that all acronyms are defined in figures and tables.

9.) *Please revise for use of patient-centered language. Please note that patient-centered language is constructed with the use of post-modified nouns (e.g. 'patients with breast cancer’ (or similar) instead of ‘breast cancer patients’) putting the person first in the sentence structure.*

**Reply:** We have revised the manuscript for use of patient-centered language.

10.) *We feel that there are too many abbreviations used throughout the manuscript, which can be a bit disruptive to the reader. Please revise the main text carefully and significantly reduce the number of abbreviations.*

**Reply:** To reduce the number of abbreviations used, we have removed the abbreviations for tissue microarray (TMA), nearest neighbor distance (NND), whole slide image (WSI), rapid case ascertainment (RCA), pancreatic ductal adenocarcinoma (PDAC), and tumor immune microenvironment (TIME).

11.) *When revising the manuscript, please keep in mind that, as a general medical journal, we aim to serve a broad audience. Therefore, your manuscript should be accessible to readers who might not be familiar with the contents or background of your study.*

**Reply:** We have ensured that the manuscript is as accessible as possible.

12.) *We note that Reviewer #3 recommended using the REMARK checklist. Please provide the completed REMARK checklist. When completing the checklist, please use section and paragraph numbers, rather than page numbers. Please add the following statement, or similar, to the Methods: ""This study is reported as per the REporting recommendations for tumour MARKer prognostic studies (REMARK) guideline (S1 Checklist)."*

**Reply:** We have provided the completed REMARK checklist below. We note that (3) in the REMARK guidelines does not apply to this study as no treatment was applied. We have included the recommended statement in the methods section (page

| **Item to be reported** | | **Paragraph** |
| --- | --- | --- |
| **INTRODUCTION** | |  |
| 1 | State the marker examined, the study objectives, and any pre-specified hypotheses. | 2 and 3 |
| **METHODS** | |  |
| *Patients* | |  |
| 2 | Describe the characteristics (e.g., disease stage or co-morbidities) of the study patients, including their source and inclusion and exclusion criteria. | 1 |
| 3 | Describe treatments received and how chosen (e.g., randomized or rule-based). | NA |
| *Specimen characteristics* | |  |
| 4 | Describe type of biological material used (including control samples) and methods of preservation and storage. | 2 |
| *Assay methods* | |  |
| 5 | Specify the assay method used and provide (or reference) a detailed protocol, including specific reagents or kits used, quality control procedures, reproducibility assessments, quantitation methods, and scoring and reporting protocols. Specify whether and how assays were performed blinded to the study endpoint. | 2 and 5 |
| *Study design* | |  |
| 6 | State the method of case selection, including whether prospective or retrospective and whether stratification or matching (e.g., by stage of disease or age) was used. Specify the time period from which cases were taken, the end of the follow-up period, and the median follow-up time. | 1 |
| 7 | Precisely define all clinical endpoints examined. | 7 |
| 8 | List all candidate variables initially examined or considered for inclusion in models. | 1,5, and 6 |
| 9 | Give rationale for sample size; if the study was designed to detect a specified effect size, give the target power and effect size. | 1 |
| *Statistical analysis methods* | |  |
| 10 | Specify all statistical methods, including details of any variable selection procedures and other model-building issues, how model assumptions were verified, and how missing data were handled. | 7 |
| 11 | Clarify how marker values were handled in the analyses; if relevant, describe methods used for cutpoint determination. | 5 and 6 |
| *Data* |  |  |
| 12 | Describe the flow of patients through the study, including the number of patients included in each stage of the analysis (a diagram may be helpful) and reasons for dropout. Specifically, both overall and for each subgroup extensively examined report the numbers of patients and the number of events. | 1 |
| 13 | Report distributions of basic demographic characteristics (at least age and sex), standard (disease-specific) prognostic variables, and tumor marker, including numbers of missing values. | 1 (Table 1) |
| **RESULTS** | |  |
| *Analysis and presentation* | |  |
| 14 | Show the relation of the marker to standard prognostic variables. | 2 and 3 |
| 15 | Present univariable analyses showing the relation between the marker and outcome, with the estimated effect (e.g., hazard ratio and survival probability). Preferably provide similar analyses for all other variables being analyzed. For the effect of a tumor marker on a time-to-event outcome, a Kaplan-Meier plot is recommended. | 5,7,8,  and 9 |
| 16 | For key multivariable analyses, report estimated effects (e.g., hazard ratio) with confidence intervals for the marker and, at least for the final model, all other variables in the model. | 5,6,10,  and 11 |
| 17 | Among reported results, provide estimated effects with confidence intervals from an analysis in which the marker and standard prognostic variables are included, regardless of their statistical significance. | 6 |
| 18 | If done, report results of further investigations, such as checking assumptions, sensitivity analyses, and internal validation. | 4,12,13,14, and 15 |
| **DISCUSSION** | |  |
| 19 | Interpret the results in the context of the pre-specified hypotheses and other relevant studies; include a discussion of limitations of the study. | 1,2,3,4, and 5 |
| 20 | Discuss implications for future research and clinical value. | 6 |

13.)  *Please confirm that the data availability, financial disclosure, and competing interest statements are in accordance with PLOS requirements, and that you provided the correct, updated versions in the online submission form. You may then remove the statements from the main text.*

**Reply:** The data availability, financial disclosure, and competing interests statements are in accordance with PLOS requirements and we will ensure the correct versions are in the online submission form and have been removed from the main text.

Editorial Comments: Abstract

1.) *Please confirm that your abstract complies with our requirements, including providing all the information relevant to this study type* [*https://journals.plos.org/plosmedicine/s/submission-guidelines#loc-abstract*](https://journals.plos.org/plosmedicine/s/submission-guidelines#loc-abstract)*.*

**Reply:** We have reviewed the guidelines linked above and updated the abstract to include any missing components.

2.)  *Please confirm that all numbers presented in the abstract are present and identical to numbers presented in the main manuscript text.*

**Reply:** All numbers in the abstract match those reported in the text.

3.) *In the last sentence of the Abstract Methods and Findings section, please describe the main limitation(s) of the study's methodology.*

**Reply:** We have updated the abstract: methods and Findings to include a sentence on the main limitation of the study.

Though these results are promising, our exploration of the tumor immune microenvironment was limited by the small number of immune markers available for our data.

4.) *In the abstract, please include the important dependent variables that are adjusted for in the analyses.*

**Reply:** We have added the important variables adjusted for (age, stage, grade, ER status, PR status, and HER2 status) to the abstract.

5.)  *“Tumor Infiltrating Lymphocytes (TILs) are prognostic in Triple-Negative Breast Cancer (TNBC), but not Estrogen Receptor (ER) positive cancers which comprise 70-80% of breast cancers.” – considering a broad medical audience, we suggest briefly explaining why TILS are not prognostic in ER+ cancers.*

**Reply:** We have updated the abstract to explain why TILs have previously not been prognostic in ER+ cancers.

6.)  *Abstract Background: The final sentence should clearly state the study question.*

**Reply:** We have updated the abstract to explicitly state the study objective in the final sentence of the Abstract Background section.

7.) *“Worse relapse-free survival (RFS) was observed for both ER+ and ER- breast cancers with low proximity and consistency of CD8+ cells.” – we suggest rephrasing to focus on better RFS for high proximity and consistency of CD8+ T-cells.*

**Reply:** We have rephrased the above sentence as suggested by the editors.

8.)  *“Among ER- breast cancers, proximity had the highest RFS hazard ratio (HR 1.84, 95% CI [1.18,2.87]).” – compared to?*

**Reply:** This is in comparison to count and consistency. We have revised the abstract to address this (page 2, line 43):

Among ER- breast cancers, proximity had the highest RFS hazard ratio (HR 1.84, 95% CI [1.18,2.87]) compared to count and consistency.

9.)  *“spatial characteristics described by proximity and consistency are consistently associated with recurrence irrespective of ER status.” – we believe that this statement might be useful to add to the Abstract findings and/or conclusions.*

**Reply:** We have added this sentence to the conclusion subsection of the Abstract.

Editorial Comments: Author Summary

1.)  *In the author summary, in the final bullet point of 'What Do These Findings Mean?', please include the main limitations of the study in non-technical language.*

**Reply:** We have included a bullet point addressing the main limitation of the study. The excerpt is given below

A key limitation of this study is that we were unable to fully interrogate immune cell interactions within the tumor due to a lack of available markers.

Editorial Comments: Methods and Results

1.) *When reporting ages, please include a unit, such as ‘years’. Please revise throughout.*

**Reply:** Where age is mentioned, we have included the unit of years.

2.) *Please include the ethics approval number.*

**Reply:** We have moved the “Statement of Ethics” section to the Methods and included the IRB approval number. The excerpt is given below:

Statement of Ethics

The study was approved by the University of North Carolina Institutional Review Board (92-0410) in accordance with U.S. Common Rule. All study participants provided written informed consent prior to study entry. This study complied with relevant ethical regulations, including the Declaration of Helsinki.

3.) *Figure 1: How was race/ethnicity defined and by whom? Why was race/ethnicity considered important in this study and what it is believed to represent [eg, are SES or genetic differences being attributed to race/ethnicity?]*

**Reply:** The Carolina Breast Cancer Study (CBCS) is unique in that it oversamples young and African American individuals. Race is self-reported by the study participants and then dichotomized into African American and Not African American. The purpose of reporting race in Table 1 is to show the demographic breakdown of the study population as required in the REMARK guidelines, as well as highlight that the overall demographics before and after quality control remain relatively unchanged.

4.)  *Please clarify at what temperatures the different IF staining steps were conducted.*

**Reply:** Incubation was performed at room temperature. The “TMA Staining and Processing” subsection of the methods has been updated to include this.

5.)  *Please note that you switch between different descriptions of CD8+ T-cells (including CD8+ cells, CD8+ T-cells, CD8+ lymphocyte). Please revise and use a consistent format.*

**Reply:** We have corrected the manuscript to only use “CD8+ T cells”.

6*.)  Please note that you switch between ‘recurrence-free survival’ and ‘relapse-free survival’. Please revise and use a consistent format.*

**Reply:** We have corrected the manuscript to only use “recurrence-free survival”.

7.)  *Please ensure that where relevant figures include 95% CIs.*

**Reply:** We have added 95% CIs to the hazard ratios in Fig4 and the spearman correlation coefficient in Fig7.

8.) *Please show graph axes beginning at zero. If this is not possible, please show a break in the axis.*

**Reply:** We have included breaks in the axis for all relevant plots.

9.) *In the Kaplan-Meier curve(s) please provide the number at risk for each time interval.*

**Reply:** We have included at risk counts in the Kaplan-Meier curve plots.

10.) *Please specify the variables controlled for in all relevant Tables.*

**Reply:** We have updated the relevant tables to include a legend listing the variables that we are controlling for.

General Editorial Requests

1.) *We ask every co-author listed on the manuscript to fill in a contributing author statement. If any of the co-authors have not filled in the statement, we will remind them to do so when the paper is revised. If all statements are not completed in a timely fashion this could hold up the re-review process. Should there be a problem getting one of your co-authors to fill in a statement we will be in contact. YOU MUST NOT ADD OR REMOVE AUTHORS UNLESS YOU HAVE ALERTED THE EDITOR HANDLING THE MANUSCRIPT TO THE CHANGE AND THEY SPECIFICALLY HAVE AGREED TO IT.*

**Reply:** All co-authors listed have completed the contributing author statement.

2.) *Please ensure that the paper adheres to the PLOS Data Availability Policy (see http://journals.plos.org/plosmedicine/s/data-availability), which requires that all data underlying the study's findings be provided in a repository or as Supporting Information. For data residing with a third party, authors are required to provide instructions with contact information for obtaining the data. PLOS journals do not allow statements supported by "data not shown" or "unpublished results." For such statements, authors must provide supporting data or cite public sources that include it.*

**Reply:** We confirm that the paper adheres to the PLOS Data Availability Policy in that we have a legitimate exception for sharing data publicly. Due to the data use agreements for CBCS, data is not publicly available. CBCS data are available upon request (<https://unclineberger.org/cbcs>).

3.) *Please review your reference list to ensure that it is complete and correct. If you have cited papers that have been retracted, please include the rationale for doing so in the manuscript text, or remove these references and replace them with relevant current references. Any changes to the reference list should be mentioned in the rebuttal letter that accompanies your revised manuscript.*

**Reply:** We have double checked and, to the best of our knowledge, there are no retracted papers included in our references. We have removed one paper that was a preprint of a recently published research article, which is also referenced.
